# Evaluating fit indices in a multilevel latent growth model with unbalanced design: a Monte Carlo study

**DOI:** 10.3389/fpsyg.2024.1366850

**Published:** 2024-05-03

**Authors:** Fan Pan, Qingqing Liu

**Affiliations:** ^1^School of Education Science, Huizhou University, Huizhou, China; ^2^Business School, Beijing Technology and Business University, Beijing, China

**Keywords:** multilevel latent growth model, unbalanced design, CFI-related fit indices, TFI-related fit indices, RMSEA-related fit indices, SRMR-related fit indices, chi-square-related fit indices

## Abstract

This study informed researchers about the performance of different level-specific and target-specific model fit indices in the Multilevel Latent Growth Model (MLGM) with unbalanced design. As the use of MLGMs is relatively new in applied research domain, this study helped researchers using specific model fit indices to evaluate MLGMs. Our simulation design factors included three levels of number of groups (50, 100, and 200) and three levels of unbalanced group sizes (5/15, 10/20, and 25/75), based on simulated datasets derived from a correctly specified MLGM. We evaluated the descriptive information of the model fit indices under various simulation conditions. We also conducted ANOVA to calculated the extent to which these fit indices could be influenced by different design factors. Based on the results, we made recommendations for practical and theoretical research about the fit indices. CFI- and TFI-related fit indices performed well in the MLGM and could be trustworthy to use to evaluate model fit under similar conditions found in applied settings. However, RMSEA-related fit indices, SRMR-related fit indices, and chi square-related fit indices varied by the factors included in this study and should be used with caution for evaluating model fit in the MLGM.

## Introduction

Social science researchers are often interested in understanding how characteristics of individuals or entities change over time (Wang et al., 2015). These characteristics could be observations about general behavior or overall academic performance, or they could be observations about specific constructs, such as depression, communication skills, attitudes toward teachers or parents, or math ability (Baumert et al., 2012). Longitudinal studies describe the changing pattern of characteristics of interest. Longitudinal studies also investigate the questions such as of how change comes about, how much change occurs, how the change process might differ across observations, and the determinants of that change over a set period.

If research questions consider both change over time and nested data, the use of Multilevel Latent Growth Model (MLGM) have been advocated as a method for analysis. MLGM, an multilevel structure equation model (SEM) extends the Latent Growth Model (LGM) model by accommodating the dependence between observations due to nested longitudinal data (Shi et al., 2019). The nested longitudinal data include repeated measures for each individual nested within the groups, thus forming a three-level structure. Based on previous research (e.g., Longford and Muthén, 1992; Linda et al., 1993), the three-level structure can be specified with a two-level model. In this two-level model, individual related parameters are estimated in the within-level model, and group related parameters are evaluated in the between-level model. MLGM can output different parameters for different levels, allowing researchers to separately study different levels.

Combining both the benefits of multilevel models and LGM, MLGM is ideally suited for addressing the research questions concerning multilevel longitudinal data (Hsu et al., 2016). A MLGM combines advantages of LGM (e.g., ability to incorporate indirect effects, complex measurement error structures, and multiple group analysis) while also correcting extent of clustering (Palardy, 2008). MLGM investigate both observations and group trajectories within one analysis. In MLGM, the individual level model and group level model have different latent intercepts and latent slopes, so individual level and group level can have different growth patterns (Rappaport et al., 2019). Further, MLGMs can include characteristics of both observation and group levels to explain the influence of various characteristics on: the change patterns of two levels, the change of measured attributes of observations within each group, and the change of all observations’ measured attributes (Hsu et al., 2016). In addition, compared to LGM, which only considers the means of measured attributes of time points, MLGM measures both the means of different times points and the means of different groups; this can assist researchers’ understanding of the overall status of the measured attributes of different groups. Muthén (1997) compared the model estimation results of MLGM, such as model fit indices and standard errors of parameters, to results of other SEM models. Compared to LGM and multilevel Confirmatory Factor Analysis, MLGM computed the best model evaluation information, indicating that MLGM was the appropriate hypothesized model for multilevel longitudinal data.

**Figure 1:**
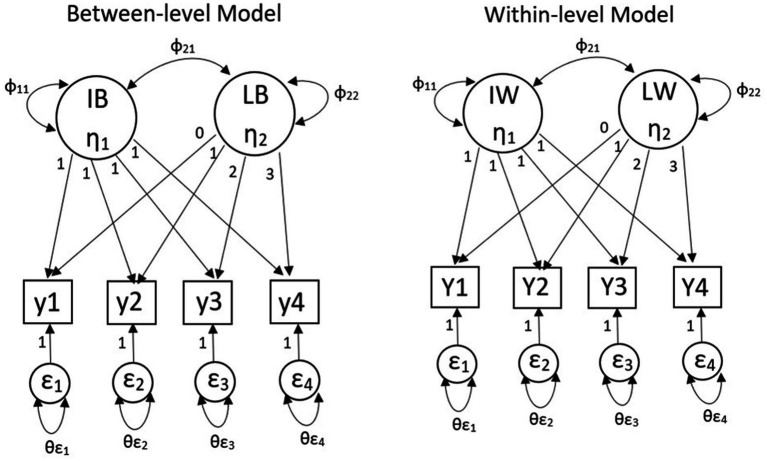
Two-level multilevel latent growth model.

Figure 1 shows a two-level linear growth MLGM with four constant growth time points. IW (η1) represents the intercept of an individual’s growth trajectory and LW (η2) represents the slope of an individual’s growth trajectory. Y1-Y4 represent four continuous outcomes for individuals, and ε1–ε4 represent the degree of deviation between the observed outcome and the expected outcome of individuals. Λ represents the factor loading for individual-level; φ represents the factor variances and covariances for individual-level; θε represents the error variances and covariances for individual-level. η is the latent variable means for individual-level. IB (η1) represents the intercept of a group’s growth trajectory and LB (η2) represents the slope of a group’s growth trajectory. Y1–Y4 represent four continuous outcomes for groups, and ε1–ε4 represent the degree of deviation between the observed outcome and the expected outcome for groups. Λ represents the factor loading for group-level; φ represents the factor variances and covariances for group-level; θε represents the error variances and covariances for group-level. η is the latent variable means for group-level. Under this condition, both group-level and individual-level’s matrices Λ will be fixed. The matrices φ and the matrix θε of both levels will be estimated. Typically, factor loadings of different levels are set to be equal to obtain unbiased parameter estimates and statistical inferences (Muthén, 1997).

The terms balanced and unbalanced are frequently encountered with longitudinal analysis approaches. A balanced design describes multilevel longitudinal data in which equal observations are planned to be measured at the different groups, whereas an unbalanced design occurs when the number of observations planned to be measured at each group is not the same. It is common to encounter an unbalanced design in empirical situations (Graham and Coffman, 2012). For example, states’ educational policies may have a general requirement for the number of students in each class or school. The students in each class or school will fluctuate around this general number. Consider that policy states the number of students in each class to be 20; however, the actual number of students could be 18, 19, 20, or 21 per classroom.

When researchers are evaluating an MLGM, typical SEM model fit indices are relied upon and commonly accepted cutoff values (or “rules of thumb”) are used for interpretation (e.g., Hu and Bentler, 1999). One common approach to evaluate MLGM is to use typical SEM model fit indices (e.g., the root mean square error of approximation [RMSEA], comparative fit index [CFI], Tucker–Lewis index [TLI], and standardized root mean square residual [SRMR]) to assess the model fit. However, there are problems with using typical SEM fit indices to judge the MLGM fit. The typical SEM fit indices are likely to be dominated by large sample size (Yuan and Bentler, 2000; Ryu and West, 2009; Hox et al., 2010) and are more sensitive to misspecification in the within-level model (Hsu et al., 2015). In MLGM, individual (i.e., within) level has much larger sample size than group (i.e., between) level. As a solution to the problems of typical SEM model fit indices when using MLGM, researchers have developed level-specific and target-specific model fit indices to detect whether the poor fit of the hypothesized MLGM comes from individual level model or group level model (Hsu et al., 2015).

There is little guidance for researchers interested in using level-specific and target-specific model fit indices for unbalanced MLGM. With balanced data, as each cluster has constantly measured attributes, one covariance and one mean structure could represent the relationship between subjects within each cluster. The mean structure of balanced data is calculated by summing the values of all individuals in the cluster to divide the fixed number of individuals (Distefano, 2016). However, as each cluster in unbalanced data do not have same numbers of subjects, one mean structure could not stand for mean structure of all clusters. Each cluster’s covariance is also different due to different numbers of measured attributes. The non-constant covariance structure within each level may cause a severe concern, especially if separate trajectories for subjects and clusters are of interest (Grund et al., 2018). Different number of subjects in each cluster may cause misspecification of mean and covariance structures for each level required by the model estimation (e.g., Laird 1988; Little and Rubin, 2019) and result in low statistical power for overall MLGM estimation (Diggle, 2002). Therefore, this study aims to fill the gaps in these literature. There has not been any study of what happens under the ‘best’ circumstances (i.e., correctly specified model). In this study, a correctly specified MLGM was simulated considering two design factors: different group sizes and unbalanced observation sizes to investigate the performance of different model fit indices under these different conditions.

## Literature review

### Investigating fit of MLGM Designs

#### Level-specific fit indices for MLGM

A partially saturated model (PS model) has been proposed to obtain the level-specific fit indices (Ryu and West, 2009). A PS model means that in an MLGM, either a within-level model or a between-level model is a saturated model. A PS model can be obtained by correlating all the observed variables and allowing all the covariances or correlations to be freely estimated at the between-level or within-level model. Ryu and West (2009) demonstrated that the PS method calculates level-specific fit indices with reasonable non-convergence rates and low Type I error rates. With a high non-convergence rate, a model fails to achieve equilibrium during analysis (Hox et al., 2010). Ryu and West (2009) indicated that PS model generated low non-convergence rate and was appropriate to generate level-specific fit indices. Using PS method, Ryu and West calculated the between-level specific fit indices (PS_B) and within-level specific fit indices (PS_W), meaning that different levels will be evaluated by different fit indices (Ryu and West, 2009; Hsu et al., 2015).

Previous literature, investigating level-specific fit indices’ performance, has been conducted for different multilevel SEMs (MSEM): multilevel confirmatory factor analysis, multilevel path models, multilevel nonlinear models, and MLGM (Ryu, 2014; Schermelleh-Engel et al., 2014; Hsu et al., 2016). Ryu and West (2009) simulated a multilevel confirmatory factor, indicating that within-level specific fit indices correctly indicated the within-level model’s poor model fit, and between-level specific fit indices successfully detect the lack of fit in the between-level model. Based on Ryu and West (2009) results, Ryu (2014) illustrated the level-specific model evaluation using empirical data and provided recommendations to researchers interested in using level specific fit indices for MSEM. As an extension of the above two studies, Hsu et al. (2016) considered the impact of intraclass correlation coefficients (ICCs) on the performance of level-specific fit indices in simulated MSEM. The ICC is defined as the ratio between group-level variance and total variance (Cohen et al., 2013). Hsu et al.’s results showed that the ICC does not significantly affect the effectiveness of between-level specific model fit indices. ICC did not influence all within-level fit indices. When ICC was very low, CFI_PS_W_ and TLI_PS_W_ can still detect the misspecification for between-level models, whereas SRMRB and RMSEA_PS_W_ did not work.

Only one study to date has concentrated on the level-specific fit indices in MLGM (Hsu et al., 2019). In line with Wu and West (2010) study, this simulation study extended Wu and West (2010) single level LGM to a two-level MLGM model with the same accelerating quadratic trajectory and time points. The estimated MLGM had five time points, and each time point was assumed to be on a standardized scale (i.e., M = 0 and SD = 1). The parameter settings were simulated based on empirical data from the Longitudinal Surveys of Australian Youth (LSAY); Following Wu et al. (2015) simulation study, Hsu et al. (2019)’s study simulated the number of clusters (NC) as, 50, 100, 200, and cluster sizes (CS) were designed into three levels, 5, 10, and 20. The results showed that CFI-and SRMR-related fit indices were not affected by small NC or CS. The RMSEA-related fit indices were likely to be influenced by small NC or CS. TLI-related fit indices needed a moderate NC (100) and CS (10). The results also indicated that within-level specific fit indices, RMSEA_PS_B_, CFI_PS_B_, and TLI_PS_B_, were not sensitive to the misspecified between-covariance structure, whereas SRMR_B_ was recommended to detect this misspecification. As for the misspecified between-mean structure, RMSEA_PS_B_, CFI_PS_B_, and TLI_PS_B_ were suggested. Among them, RMSEA_PS_B_ was recommended as it was found to be more sensitive to detecting misspecification.

### Target specific fit indices for MLGM

In addition to level-specific fit indices, our research also evaluated the performance of target-specific fit indices. Target-specific fit indices for MLGM examine whether the misspecification comes from the covariance structure or the mean structure of between-level or within-level model (DiStefano et al., 2013). Hsu et al. (2019) extended the investigation of target-specific fit indices’ performance from the context of LGM to MLGM. The authors outlined a practical way to compute the target-specific fit indices for the between-level covariance structure fit indices and the between-level mean structure fit indices. The target-fit indices for MLGM only need to be estimated at the between-level model. Because fixing the means of growth factors at zero, the misspecifications of the whole MLGM could only be attributed to the within-covariance structure (Muthén, 1997). The fit indices for between-level covariance structure (T_S_COV) include χ^2^_T_S_COV_, RMSEA_T_S_COV_, CFI_T_S_COV_, TLI_T_S_ COV_, and SRMR_T_S_COV_, and the fit indices for the between-level mean structure (T_S_MEAN) has χ^2^_T_S_ Mean_, RMSEA_T_S_ Mean_, CFI_T_S_Mean_, TLI_T_S_Mean_, and SRMR_T_S_Mean_.

Based on Wu and West (2010) and Ryu and West (2009) research, Hsu et al. (2019) generated T_S_MEAN fit by saturating the within-level model and the covariance structure of the between-level model. T_S_COV fit indices were created by saturating the within-level model and the mean structure of the between-level model. The researchers studied the influence of the sample size, cluster size, and type of misspecification on the sensitivity of target-specific fit indices for MLGM. The results indicated that RMSEA_T_S_COV_, CFI_T_S_COV_, and TLI_T_S_ COV_ showed higher sensitivity to misspecified between-variance structure than RMSEA_PS_B_, CFI_PS_B_, and TLI_PS_B_. In addition, the RMSEA_T_S_COV_ yielded a higher sensitivity than the other two fit indices. χ^2^_T_S_COV_ is also favored because of its high statistic power using for different sample size conditions. SRMR_T_S_COV_ is not recommended when the cluster size is less than 5. As for a misspecified between-mean structure, RMSEA_T_S_ Mean_, CFI_T_S_Mean_ and TLI_T_S_Mean_ did not show a higher sensitivity than RMSEA_PS_B_, CFI_PS_B_, and TLI_PS_B_. Hsu et al. (2019) recommended researchers use RMSEA_PS_B_, CFI_PS_B_, and TLI_PS_B_ to detect misspecified between structures. Both SRMR_T_S_mean_ and SRMR_B_ are not recommended because they had means and variances close to 0.

### Recommended fit indices for each data type

**Table:** 

Fit indices		Different multilevel SEMs	Sample size (<10)	Structure	Low ICC (<0.5)
Level-specific fit indices	RMSEA_PS_B_	multilevel confirmatory factor analysis, multilevel path models, multilevel nonlinear models, balanced MLGM	No Small Sample	Detect misspecified between-mean (most recommend)	Did not work
	RMSEA_PS_W_	multilevel confirmatory factor analysis, multilevel path models, multilevel nonlinear models, balanced MLGM	No Small Sample	Not sensitive	unrelated
	CFI_PS_B_	multilevel confirmatory factor analysis, multilevel path models, multilevel nonlinear models, balanced MLGM	Yes Small Sample	Detect misspecified between-mean (recommend)	work
	CFI_PS_W_	multilevel confirmatory factor analysis, multilevel path models, multilevel nonlinear models, balanced MLGM	Yes Small Sample	Not sensitive	unrelated
	TLI_PS_B_	multilevel confirmatory factor analysis, multilevel path models, multilevel nonlinear models, balanced MLGM	Moderate Sample	Detect misspecified between-mean (recommend)	work
	TLI_PS_W_	multilevel confirmatory factor analysis, multilevel path models, multilevel nonlinear models, MLGM	Moderate Sample	Not sensitive	unrelated
	SRMR_B_	multilevel confirmatory factor analysis, multilevel path models, multilevel nonlinear models, MLGM	Yes Small Sample	Detect misspecified between-covariance (not recommend)	Did not work
	SRMR_W_	multilevel confirmatory factor analysis, multilevel path models, multilevel nonlinear models, MLGM	Yes Small Sample	Not sensitive	unrelated
Target-specific fit indices	RMSEA_T_S_COV_	LGM, balanced MLGM	Not influence	Detect Misspecified between-covariance (recommend)	unrelated
	RMSEA_T_S_Mean_	LGM, balanced MLGM	Not influence	Detect misspecified between-mean	unrelated
	CFI_T_S_COV_	LGM, balanced MLGM	Not influence	Detect Misspecified between-covariance	unrelated
	CFI_T_S_Mean_	LGM, balanced MLGM	Not influence	Detect misspecified between-mean	unrelated
	TLI_T_S_COV_	LGM, balanced MLGM	Not influence	Detect Misspecified between-covariance	unrelated
	TLI_T_S_ Mean_	LGM, balanced MLGM	Not influence	Detect misspecified between-mean	unrelated
	SRMR_T_S_COV_	LGM, balanced MLGM	No Small Sample	Not sensitive	unrelated
	SRMR_T_S_Mean_	LGM, balanced MLGM	Not influence	Not sensitive	unrelated

### Unbalanced design for MLGM

As researchers can rely on level-specific and target-specific model fit indices to judge an MLGM model’s acceptability, testing if the level-specific and target-specific fit indices perform acceptably under MLGM with an unbalanced design is needed. Previous studies investigating the level-specific and target-specific model fit indices for MLGM have only examined a balanced design. Few studies concerning the MLGM model fit when unbalanced designs are present. Studies have used the effect of estimation direct maximum likelihood (direct ML) to address unbalanced issues in MLGM (Ryu and West, 2009). Direct ML conceptualizes the unbalanced design as a form of missing data. However, direct ML can only provide traditional model fit indices for MLGM and could not output level-specific and target-specific model fit indices.

For a balanced MLGM with G balanced groups, each group has n observations. The total sample size N equals nG. The MLGM defines the within group covariance matrix as SPW and the between group covariance matrix as S*B. The formulas for the SPW & S*B covariance matrices are:


$$SPW=\frac{\sum_{g}^{G}\sum_{i}^{n}\left(Y_{gi}-{\bar{Y}}_{g}\right){\left(Y_{gi}-{\bar{Y}}_{g}\right)}^{\prime }}{N-G}$$

$$S*B=\frac{\sum_{n}^{G}n\left(\bar{Y}-{\bar{Y}}_{g}\right){\left(\bar{Y}-{\bar{Y}}_{g}\right)}^{\prime }}{G-1}.$$

In the above two equations, $Y_{gi}$ represents for the response for each observation, ${\bar{Y}}_{g}$ represents the mean response of n observations in each group, and $\bar{Y}$ indicates for the mean response of all N observations in the data.

In an unbalanced MLGM situation, as groups have unequal numbers of individuals, SPW may still represent the within group covariance matrix because the SPW formula directly pools together all observations, regardless of group size. S*B, however, cannot represent the covariance matrix for each group because each group could have a distinct group size, n. Different S*B matrices will be calculated for each group. In this way, the aggregate covariance for unbalanced multilevel data no longer represents sufficient statistics for model estimation and may cause problems for model estimation.

Although unbalanced multilevel longitudinal data is common with many educational research applications, there is not yet a study that has investigated the influence of unbalanced multilevel longitudinal data on model fit indices of MLGM. Hox and Maas (2001) simulated an unbalanced multilevel data at one time point to investigate the performance of a multilevel confirmatory factor model with unbalanced data. The results indicated that unbalanced data had little impact on the accuracy of parameter estimates of the within level model. However, for the between level, the variances of model fit indices tended to be underestimated, so the standard errors of parameter estimates were too small to be accepted. As the Hox and Maas (2001)’ investigation conducted with model at only one time point, results will not improve in a MLGM situation. Model estimation for MLGM, including model fit, parameters estimation, and standard errors, may be substantially biased if the unbalanced nature is not considered.

The following research questions were examined using a Multilevel Latent Growth Model with an unbalanced design. This study examined the performance of different level-specific and target-specific model fit indices when evaluating unbalanced MLGM with different sampling errors

(1) How are level-specific and target-specific fit indices impacted by sampling error and unbalanced design?
(2) Do the level-specific and target-specific fit indices demonstrate reasonable sensitivity to sampling error and unbalanced design?

## Methods

### Population model

A Monte Carlo study was performed to evaluate the performance of both level-specific fit indices (χ^2^_PS_B_, RMSEA_PS_B_, CFI_PS_B_, TLI_PS_B_, SRMRB, χ^2^_PS_W_, RMSEA_PS_W_, CFI_PS_W_, TLI_PS_W_, SRMR_W_) and target-specific fit indices (χ^2^_T_S_COV_, RMSEA_T_S_COV_, CFI_T_S_COV_, TLI_T_S_COV_, SRMR_T_S_COV_, χ^2^_T_S_MEAN_, RMSEA_T_S_Mean_, CFI_T_S_Mean_, TLI_T_S_ Mean_, and SRMR_T_S_Mean_) in a two-level correctly specified MLGM (Jiang, 2014). The design factors include the number of groups and unbalanced group sizes.

Based on previous research, parameter settings from the LSAY (Longitudinal Study of American Youth) was used to simulate the correctly specified MLGM model (Hsu et al., 2019). The parameters used for the population model are based on one MLGM study of LSAY, which contains 3,102 students from grade 7 to grade 11 nested within 52 schools (Hsu et al., 2019). In line with previous MLGM simulation studies (Wu and West, 2010), a five-wave MLGM model was measured in this research. The five-time points, denoted as V1–V5, were assumed to be continuous data distributed on the standardized scale (i.e., Mean = 0 and SD = 1). The intraclass correlation coefficients (ICCs) of five-time points ranged from 0.15 to 0.19, showing that cluster-level should not be ignored in this MLGM study (Hox et al., 2010). In the between-level model, the parameter settings for the mean structure and covariance structure are presented in matrices αB and ΦB, and mean structure and covariance structure in within model are presented in matrices αW and ΦW.


$$\alpha B=\left[\begin{array}{c} 49.96\\ \;4.32\\ -0.13. \end{array}\right]$$

$$\alpha W=\left[\begin{array}{c} 0\\ 0\\ 0 \end{array}\right]$$

$$\Phi B=\left[\begin{array}{ccc} 16.2 & 2.82 & 0\\ 2.82 & 0.61 & 0\\ 0 & 0 & 0.02 \end{array}\right]$$

$$\Phi w=\left[\begin{array}{ccc} 71.45 & 6.76 & 0\\ 6.76 & 14.76 & 0\\ 0 & 0 & 0.070 \end{array}\right]$$

According to Wu and West (2010) in the SEM framework, the general population quadratic model for the population does not consider the covariance between the intercept and slope and covariance between linear and slope. In this way, we set the nondiagonal values in the matrix at both between-level and within-level be zero for simplicity.

The error variances for five-time points of between level model are set to 11.91, 15.25, 10.32, 12.59, and 1.93 and are uncorrelated over time. The error variances for five-time points of within level model are set to 1.80, 1.28, 0.06, 0.54, and 0.31, and these scores are also uncorrelated over time (Kwon, 2011).

The estimation of all the population models was carried out in Mplus 7.11 (Muthén and Muthén, 2017), using maximum likelihood estimation with robust standard errors (ESTIMATOR = MLR). The maximum number of iterations were set to 100 (ITERATIONS = 100) with 95 convergence criterion set to 0.000001 (CONVERGENCE = 0.000001). MLR are robust to non-normality and non-independence of observations when used with TYPE = COMPLEX (Muthén and Muthén, 2017). Our simulated datasets contain small sample sizes, which were non-normal samples. The students of simulated datasets are nested within each cluster, meaning the datasets are non-independence. MLR was the appropriate estimator for Mplus (Pornprasertmanit et al., 2013).

### Design conditions: NG and unbalanced GS

NG conditions were based on Wu et al. (2015) studies, and set at 50, 100, and 200. To maximize the effect of imbalance, the group sizes were chosen to be as different as possible. The highest number 200 conforms to Boomsma’s (1983) recommended lower limit for achieving good maximum likelihood estimates with normal data. The lower values 50 and 100 have been chosen because, in empirical multilevel modeling, it is hard to collect data from as many as 200 groups (Hox and Maas, 2001).

As with Hox and Maas (2001) simulation study and the regression rule of thumb for multilevel research, each predictor requires at least 10 observations (Bryk and Raudenbush, 1992). The averages of unbalanced CS conditions are manipulated into three levels, 10, 20, and 50. Unbalanced data were simulated as follows (Grilli and Rampichini, 2011). In each level, we employ two distinct group sizes, with exactly half the groups being small and the other half being large. I assigned small to half of the groups and large to the other half. The two numbers (large and small) were computed under the restriction that the coefficient of variation $\frac{1}{n}*\sqrt{\text{Var}\left(\text{nj}\right)}$ is approximately 0.5 (Grilli and Rampichini, 2011). The coefficient of variation indicates the degree of imbalance of the design, and 0.5 is a moderate degree of imbalance. We choose a moderate imbalance as our GS is relative small, and we do not want extremely small unbalanced influence the evaluation outcomes. The large group size is three times as large as the small group size. For unbalanced GS average is 10, small size is 5, and large sample size is 15; For unbalanced GS average is 20, small size is 10, and large sample size is 30; For unbalanced GS average is 50, small size is 25, and large sample size is 75. These three levels of CS range are also consistent with two large-scale national educational databases: the Early Childhood Longitudinal Study (Tourangeau et al., 2009) and the Early Childhood Longitudinal Study (Tourangeau et al., 2015).

In this way, we have 9 total simulation conditions. Based on the recommendation that 1,000–5,000 replication is required to produce a stable result in Monte Carlo studies (Mundform et al., 2011), 1,000 complete datasets based on population model were generated for each simulation condition. SAS 9.4 was used to simulate the datasets.

### Data analysis and outcomes

First we examined the descriptive information for model fit indices under different design factors. We also generated box plots to show the distribution of model fit indices under design factors. The second part of the analysis evaluated the sensitivity of both level-specific and target-specific model fit indices to different design factors. ANOVA with an individual model fit index’s values as the dependent variables were conducted to evaluate influence of design factors. For ANOVA, we calculated the effect size, eta-squared (η2), by dividing the Type III sum-of-square attributable to each design factor or the interaction between factors by the corrected total sum-of-square. η2 describes the proportion of the variability accounted for a particular design factor or interaction effect term. In this study, each simulation condition has the same number of simulated datasets, resulting in orthogonal design factors. In this way, the Type III sum-of-squares from different factors were additive and non-overlapping, meaning the η2 of each design factor could be calculated separately without considering other factors. Following Cohen (1988) study, we considered a moderate η2 of 0.0588 to identify practically significant design factors for the fit indices’ values. Note that when a fit index had a standard deviation close to 0, the impact of design factors on the fit index were trivial, even though the η2 s were larger than 0.0588. As for our analysis, when fit indices have extremely low variability, we regarded design factors do not affect the fit indices.

### Calculate level-specific and target-specific fit indices

χ^2^ is commonly used because it is easier to compute than other model fit indices. It can also be used with categorical data and to check the if there is a “difference” between different groups of participants. Deviations from normality and small sample may result in poor χ^2^ value even though the model is appropriately specified (Goos et al., 2013). For well-fitted models, cut off values of SRMR are supposed to be less than 0.05, and values as high as 0.08 are sometimes also deemed acceptable (Bentler and Dudgeon, 1996; Chou et al., 1998). However, when there are many parameters in the model and large sample sizes, SRMR also gives acceptable values even though the hypothesized model does not fit the dataset (Boulton, 2011). Unlike χ^2^ and SRMR, RMSEA is not affected by the sample size, which means that RMSEA can still evaluate the model with small sample sizes (Clarke et al., 2008). Even though TLI is not affected significantly by the sample size, the TLI value can show poor fit when other fit indices are pointing toward good fit in models where small samples are used (Bentler, 1990; Boulton, 2011). CFI is relatively independent of sample size and yields better results for studies with a small sample size (Chen et al., 2012).

χ^2^_PS_B_ can be obtained by specifying a hypothesized between model and saturating the within model (Hox et al., 2010). A saturated model can be seen as a just-identified model with zero degrees of freedom, and thus has a χ^2^ test statistic equal to zero. As a result, χ^2^_PS_B_ only reflect the model fit of the hypothesized between model (Hox et al., 2010). After χ^2^_PS_B_ is obtained, between-level specific fit indices: RMSEA_PS_B_, CFI_PS_B_, TLI_PS_B_, SRMR_B_ can be computed, because these fit indices are calculated based on χ2_PS_B_. In the same way, within-level specific fit indices (χ^2^_PS_W_, RMSEA_PS_W_, CFI_PS_W_, TLI_PS_W_, SRMR_W_) can be also be computed. Target-specific fit indices for the mean structure only (χ^2^_T_S_MEAN_, RMSEA_T_S_Mean_, CFI_T_S_Mean_, TLI_T_S_ Mean_, and SRMR_T_S_Mean_) can be derived by saturating the covariance structure of the between level of MLGM, whereas target-specific fit indices for the covariance structure only (χ^2^_T_S_COV_, RMSEA_T_S_COV_, CFI_T_S_COV_, TLI_T_S_COV_, and SRMR_T_S_COV_) can be derived by saturating the mean structure of the between level of MLGM.

## Results

Under the various design conditions, the convergence rates over the 1,000 replications were 100% across all cells in the design. Thus, even under the smallest sample size [number of groups (NG) = 50, with an unbalanced group size (GS) = 5], the analysis was unlikely to encounter convergence problems. Results were summarized across all replications. Traditional cutoff criteria of the fit indices used with typical SEM studies (e.g., RMSEA <0.06; CFI and TLI >0.95; SRMR <0.08; Hu and Bentler, 1999) were examined with simulated model to determine if these recommended levels were able to accurately identify correct models across different number of groups and unbalanced group sizes. Table 1 summarizes the means and standard deviations of all fit indices.

**Table 1:** Descriptive statistics of model fit indices by NG and unbalanced GS for the accelerating growth trajectory.

Fit index	NG	50	50	50	100	100	100	200	200	200
	GS	5/15	10/30	25/75	5/15	10/30	25/75	5/15	10/30	25/75
χ^2^test statistics										
χ^2^PS_B	Mean	29.38	14.76	5.13	11.08	5.16	4.37	5.30	4.31	4.01
	SD	240.63	68.62	8.89	46.92	4.42	3.14	4.85	3.01	2.83
χ^2^PS_W	Mean	14.16	16.31	4.31	14.40	4.62	4.03	4.92	4.17	3.96
	SD	32.34	164.39	3.34	93.74	3.77	2.91	3.94	3.03	3.01
χ^2^T_S_COV	Mean	137.53	100.67	73.21	87.20	102.43	131.03	121.63	179.80	249.44
	SD	729.46	532.83	23.19	58.96	38.77	28.76	39.91	36.79	39.47
χ^2^T_S_Mean	Mean	80.33	26.39	23.57	22.75	25.71	35.74	27.49	40.92	62.85
	SD	828.27	62.94	9.691	19.68	11.06	10.92	12.00	12.18	14.44
RMSEA-related fit indices										
RMSEAPS_B	Mean	0.054	0.027	0.008	0.027	0.010	0.005	0.010	0.005	0.003
	SD	0.100	0.046	0.012	0.035	0.012	0.007	0.013	0.007	0.004
RMSEAPS_W	Mean	0.045	0.024	0.007	0.027	0.008	0.004	0.009	0.005	0.003
	SD	0.058	0.052	0.009	0.045	0.011	0.006	0.012	0.007	0.004
RMSEAT_S_COV	Mean	0.144	0.098	0.061	0.102	0.081	0.059	0.089	0.078	0.059
	SD	0.128	0.061	0.010	0.032	0.014	0.007	0.015	0.008	0.005
RMSEAT_S_Mean	Mean	0.074	0.044	0.029	0.042	0.035	0.028	0.037	0.034	0.028
	SD	0.124	0.029	0.009	0.022	0.011	0.005	0.011	0.006	0.004
CFI-related fit indices										
CFIPS_B	Mean	0.995	0.999	1.000	0.999	1.000	1.000	1.000	1.000	1.000
	SD	0.035	0.008	0.000	0.005	0.000	0.000	0.000	0.000	0.000
CFIPS_W	Mean	0.998	0.999	1.000	0.999	1.000	1.000	1.000	1.000	1.000
	SD	0.007	0.016	0.000	0.010	0.000	0.000	0.000	0.000	0.000
CFIT_S_COV	Mean	0.977	0.990	0.996	0.990	0.994	0.997	0.993	0.994	0.997
	SD	0.061	0.033	0.001	0.006	0.002	0.001	0.002	0.001	0.001
CFIT_S_Mean	Mean	0.991	0.998	0.999	0.998	0.999	0.999	0.999	0.999	0.999
	SD	0.061	0.008	0.000	0.002	0.001	0.000	0.001	0.000	0.000
TLI-related fit indices										
TLIPS_B	Mean	0.972	0.994	1.000	0.996	1.000	1.000	1.000	1.000	1.000
	SD	0.254	0.042	0.002	0.027	0.001	0.000	0.002	0.001	0.000
TLIPS_W	Mean	0.989	0.994	1.000	0.994	1.000	1.000	1.000	1.000	1.000
	SD	0.035	0.079	0.001	0.051	0.001	0.000	0.001	0.001	0.000
TLIT_S_COV	Mean	0.924	0.970	0.990	0.973	0.982	0.990	0.979	0.983	0.990
	SD	0.403	0.147	0.003	0.018	0.006	0.002	0.007	0.003	0.002
TLIT_S_ Mean	Mean	0.959	0.994	0.997	0.995	0.996	0.998	0.996	0.997	0.998
	SD	0.460	0.022	0.001	0.006	0.002	0.001	0.002	0.001	0.001
SRMR-related fit indices										
SRMRB	Mean	0.012	0.008	0.005	0.008	0.005	0.004	0.005	0.003	0.002
	SD	0.009	0.005	0.003	0.005	0.003	0.002	0.003	0.002	0.001
SRMRW	Mean	0.004	0.002	0.001	0.002	0.002	0.001	0.002	0.001	0.001
	SD	0.002	0.001	0.001	0.001	0.001	0.001	0.001	0.001	0.000
SRMRT_S_COV	Mean	0.172	0.183	0.193	0.170	0.184	0.193	0.168	0.183	0.193
	SD	0.042	0.027	0.019	0.029	0.019	0.013	0.021	0.012	0.009
SRMRT_S_Mean	Mean	0.067	0.070	0.072	0.066	0.070	0.071	0.068	0.072	0.072
	SD	0.035	0.030	0.021	0.028	0.021	0.015	0.021	0.014	0.011

RMSEA, root mean square error of approximation. CFI, comparative fit index. TLI, Tucker–Lewis index. SRMR, standardized root mean square residual. Subscripted PS, partially saturated model method. Subscripted TS, target-specific fit indices. Subscripted B, between-level model. Subscripted W, within-level model. Subscripted COV, fit index for evaluating between-covariance structure. Subscripted MEAN, fit index for evaluating between-mean structure.

Descriptive statistics in Table 1 showed that when NG increased from 50 to 200 and unbalanced GS increased from 5/15 to 25/75, χ^2^_PS_B_ and χ^2^_PS_W_ mean values and standard deviation values decreased. Both indices showed that the average estimated χ^2^_PS_B_ and χ^2^_PS_W_ approached the expected value (i.e., 4 degrees of freedom) when NG = 50 and unbalanced GS = 25/75. A total sample size over 1,250 was necessary for χ^2^PS_w and χ^2^_PS_B_ to appropriately identify correct between-level and within-level models. For the χ^2^_T_S_COV_ and χ^2^_T_S_MEAN_, the descriptive values mean of χ^2^_T_S_COV_ and χ^2^_T_S_MEAN_ did not approach acceptable model fit when total sample size increased. All means of RMSEA_PS_B_ and RMSEA_PS_W_ approached acceptable model fit (i.e., <0.06) and standard deviation values decreased as sample size increased. Also, all RMSEA_T_S_COV_ mean values did not approach levels indicative of acceptable model under the tested sample sizes. For the RMSEA_T_S_MEAN_, values yielded an acceptable model, except under the smallest sample size condition (NG = 50, unbalanced GS = 5/15). The standard deviation of RMSEA_T_S_COV_ and RMSEA_T_S_MEAN_ decreased as sample sizes increased. Means of CFI-and TLI-related fit indices were indicative of good model fit (i.e., >0.95) under almost all simulation conditions. There was only one mean value, TLI_T_S_COV_, that yielded a value under the stated cutoff (NG of 50, unbalanced GS of 5/15). All means of SRMR_B_, SRMRw, and SRMR_T_S_MEAN_ produced values indicating acceptable model fit (i.e., <0.08); however, means of SRMR_T_S_COV_ were larger, approaching the cutoff of poor model fit under all simulation conditions. The standard deviations values of SRMRB and SRMRw were close to a value 0 and standard deviation of SRMR_T_S_COV_ and SRMR_T_S_MEAN_ were also small.

### ANOVA review of conditions

**Figure 2:**
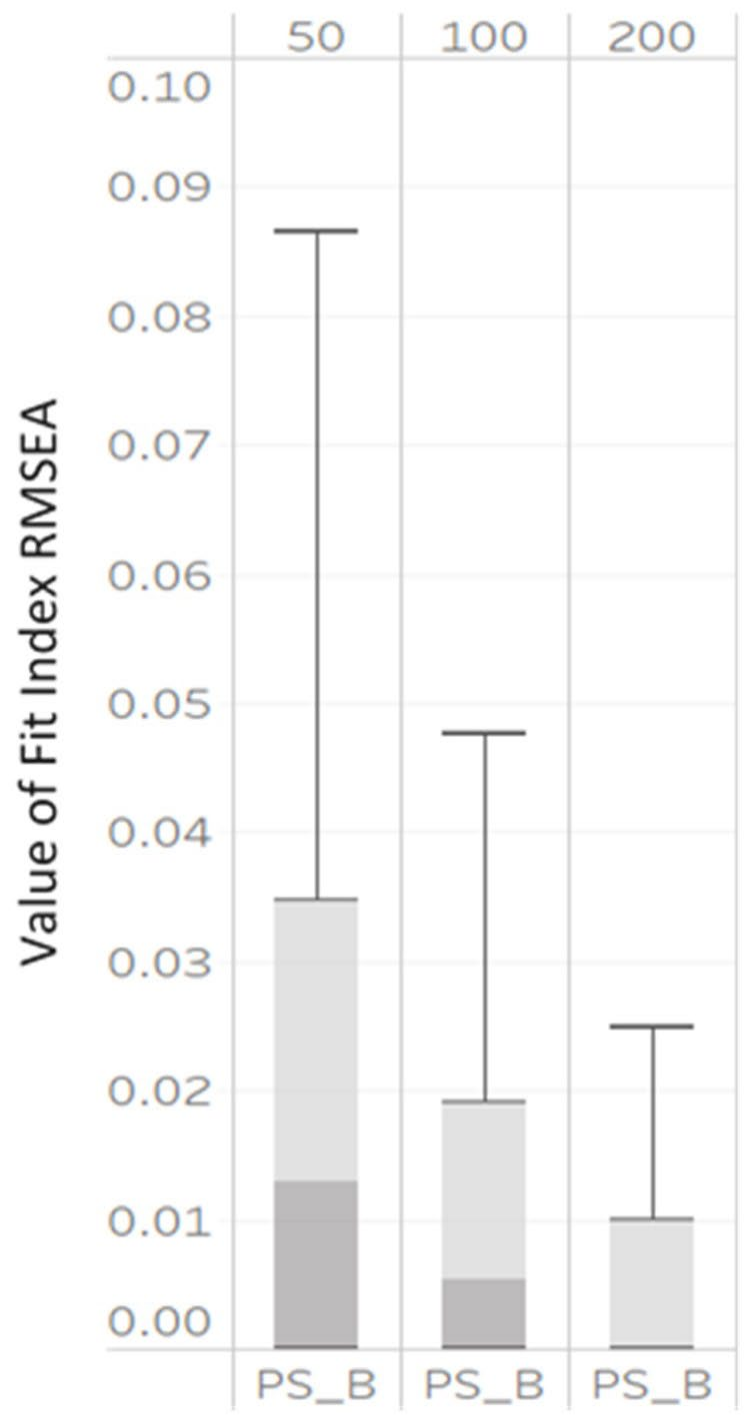
Box plot of RMSEAPS_B values correctly specified MLGM models by NG (50, derived from 100, and 200).

**Figure 3:**
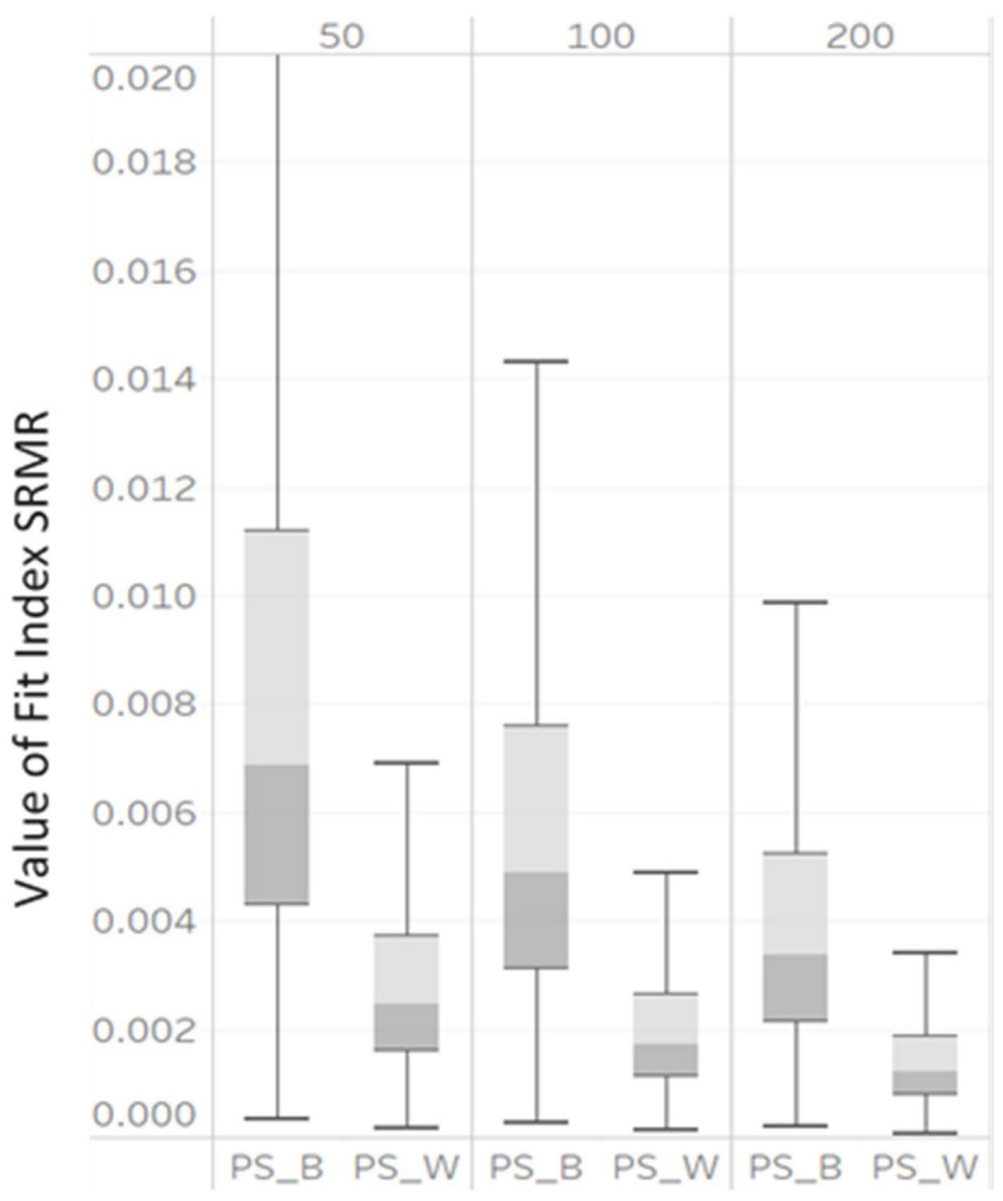
Box plot of SRMRB and SRMRW values derived from correctly specified MLGM models by NG (50, 100, and 200).

To determine factors that affected model fit indices, a three-way ANOVA with 3 (NG: 50, 100, and 200) x 3 (unbalanced GS: 5/15, 10/30, and 25/75) levels was conducted, with each fit index as the outcome variable. The η2 for each design factor is presented in Table 2. We provide a visual representation of the influential design factors on the fit indices’ values with boxplots in Figures 2, 3, respectively.

**Table 2:** η2 values from ANOVA design by fit index.

Dependent variables	Number of group (NG)	Unbalanced group size (GS)
χ^2^test statistics		
χ^2^PS_B	0.00	0.00
χ^2^PS_W	0.00	0.00
χ^2^T_S_COV	0.00	0.00
χ^2^T_S_Mean	0.00	0.00
RMSEA-related fit indices		
RMSEAPS_B	0.06	0.07
RMSEAPS_W	0.05	0.06
RMSEAT_S_COV	0.02	0.06
RMSEAT_S_Mean	0.03	0.06
CFI-related fit indices		
CFIPS_B	0.00	0.00
CFIPS_W	0.00	0.00
CFIT_S_COV	0.02	0.02
CFIT_S_Mean	0.00	0.01
TLI-related fit indices		
TLIPS_B	0.00	0.00
TLIPS_W	0.00	0.00
TLIT_S_COV	0.00	0.00
TLIT_S_ Mean	0.00	0.00
SRMR-related fit indices		
SRMRB	0.13	0.14
SRMRW	0.16	0.22
SRMRT_S_COV	0.00	0.02
SRMRT_S_Mean	0.00	0.01

RMSEA, root mean square error of approximation. CFI, comparative fit index. TLI, Tucker–Lewis index. SRMR, standardized root mean square residual. Subscripted PS, partially saturated model method. Subscripted TS, target-specific fit indices. Subscripted B, between-level model. Subscripted W, within-level model. Subscripted COV, fit index for evaluating between-covariance structure. Subscripted MEAN, fit index for evaluating between-mean structure. Highlighted (gray shaded cells) η2 ≥ 0.0.

Based on η2 values in Table 2, only three indices: RMSEA_PS_B_, were impacted by NG, with η2 values of 0.06, 0.13, and 0.26, respectively. Further, the boxplots in Figure 2 showed that the RMSEA_PS_B_ computed under all simulation conditions were large at lower sample sizes. As the NG increased, the median RMSEA_PS_B_ decreased with values of 0.12, 0.04, to 0.001 associated with NG levels of 50, 100, and 200, respectively. The interquartile ranges also became smaller, indicating the values of RMSEA_PS_B_ were less dispersed.

SRMR_B_ and SRMR_W_ also demonstrated large variability under the simulation conditions (shown in Figure 3). As the NG varied from 50, 100, to 200, the median SRMR_B_ decreased from 0.007, to 0.005, to 0.0035 and the median SRMR_W_ decreased from 0.0025, to 0.0018, to 0.001. The interquartile ranges of all SRMR_B_ and SRMR_W_ also became smaller.

**Figure 4:**
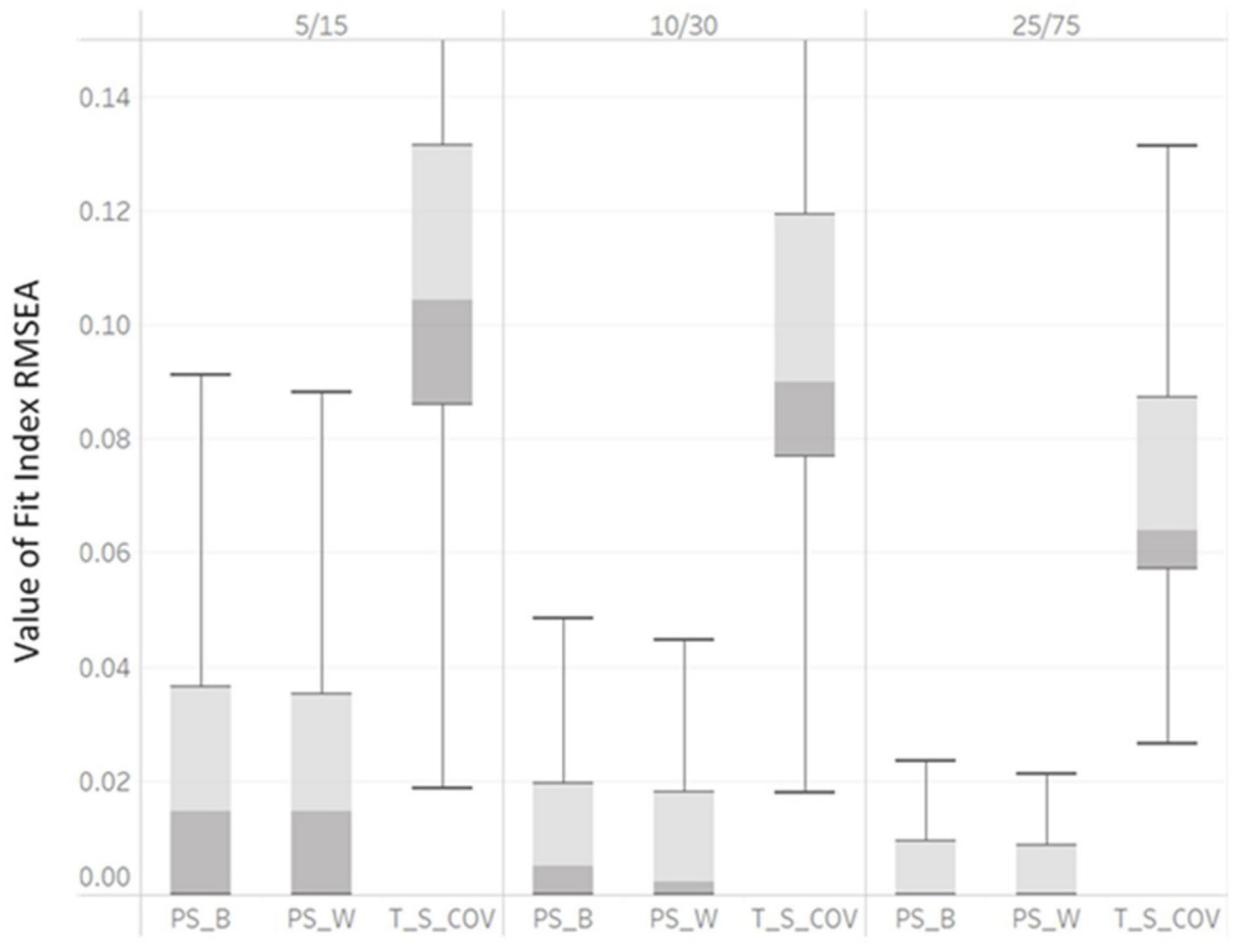
Box plot of RMSEAPS_B, RMSEAPS_W, and RMSEAT_S_COV values derived from correctly specified MLGM Models by unbalanced GS (5/15, 10/30, and 25/75).

In Table 2, η2 indicated that RMSEA_PS_B_, RMSEA_PS_W_, RMSEA_T_S_COV_, SRMR_B_ and SRMR_W_ were impacted by unbalanced GS (η2 = 0.07, 0.06, 0.06, 0.14 and 0.22). As the unbalanced GS varied from 5/15, 10/30, to 25/75, the median RMSEA_PS_B_ would show poor fit at the smallest level (0.08) but not at later levels (0.04, 0.001 for 10/30 and 25/75, respectively). As the unbalanced GS increased, the median RMSEA_PS_W_ decreased with values of 0.08, 0.01, to 0.001 and the median RMSEA_T_S_COV_ decreased with values of 0.12, 0.09, to 0.064 with unbalanced GS levels of 5/15, 10/30, and 25/75, respectively. Box plots are presented in Figure 4. The interquartile ranges of all RMSEA_PS_B_, RMSEA_PS_W_, and RMSEA_T_S_COV_ also smaller, indicating the values of RMSEA_PS_B_, RMSEA_PS_W_, and RMSEA_T_S_COV_ were less dispersed. Both the η2 and boxplots showed that RMSEA_PS_B_, RMSEA_PS_W_, and RMSEA_T_S_COV_ were affected by factor unbalanced GS and may indicate values indicating poor model fit for a correctly specified model fit.

**Figure 5:**
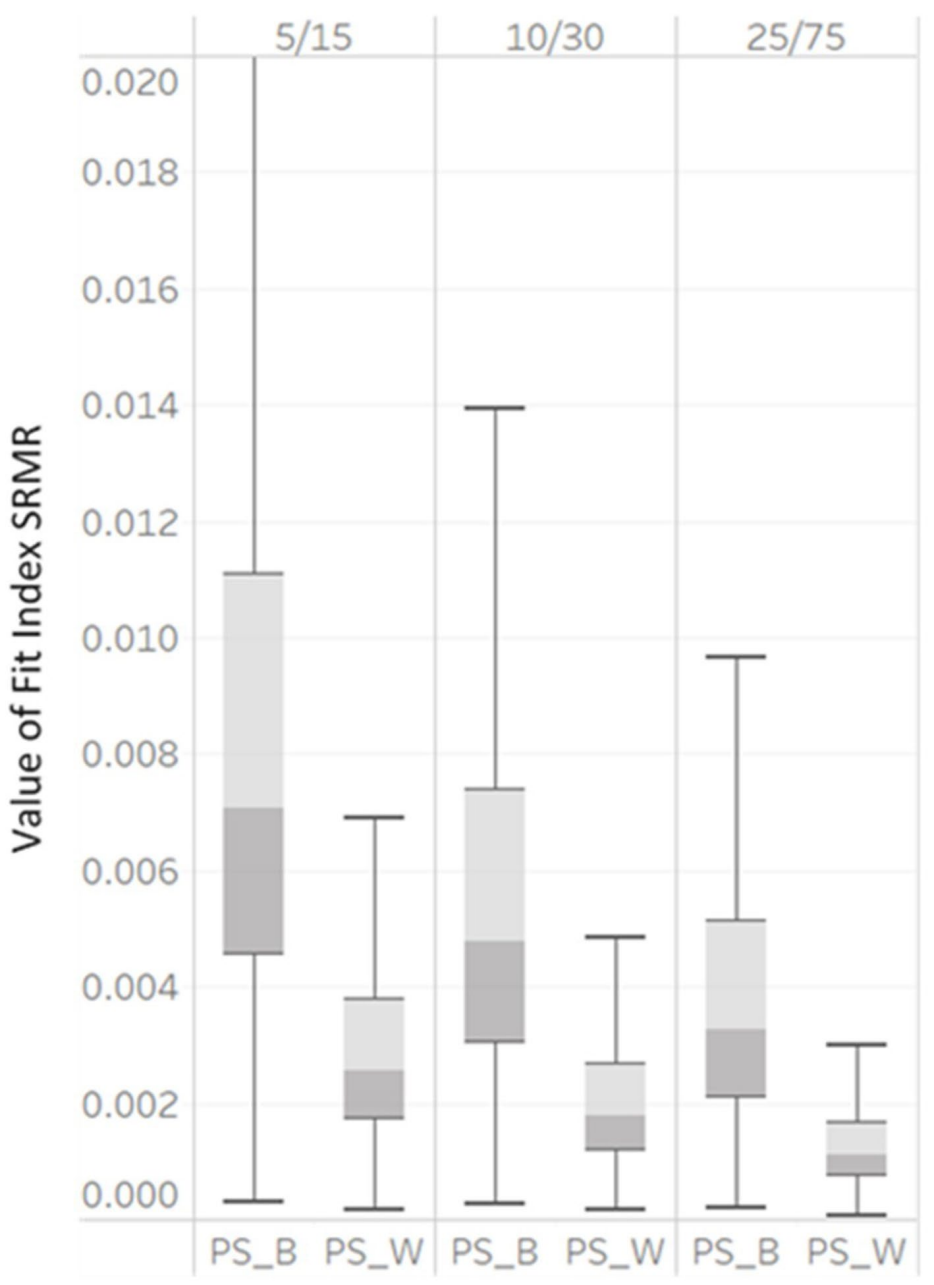
Box plot of SRMRB and SRMRW values derived from correctly specified MLGM models by unbalanced GS (5/15, 10/30, and 25/75).

Figure 4 demonstrated the variabilities of SRMR_B_ and SRMR_W_ computed under all simulation conditions were large. As the unbalanced GS increased, the median SRMR_B_ decreased (with values of 0.007, 0.005, to 0.0032) as did the median SRMRW decreased (values of 0.0024, 0.0018, to 0.001) for unbalanced GS levels of 5/15, 10/30, to 25/75, respectively. The interquartile ranges of all SRMR_B_ and SRMR_W_ also became smaller, indicating the values of SRMR_B_ and SRMR_W_ were less dispersed. Both the η2 and boxplots showed SRMR_B_ and SRMR_W_ were affected by factor unbalanced GS. With increase of the unbalanced GS, SRMR_B_ and SRMR_W_ showed lower model fit values (Figure 5).

## Discussion

In practice, given that researchers are not aware if the model is correctly specified, researchers are recommended to use χ^2^_PS_B_ and χ^2^_PS_W_ when the sample size is large enough (NG > 100 and unbalanced GS > 10/30). Based on our findings, researchers need to be aware that using χ^2^_T_S_COV_ and χ^2^_T_S_MEAN_ will cause the over-rejection of correctly specified unbalanced MLGM. Based on these findings, χ^2^_T_S_COV_ and χ^2^_T_S_MEAN_ are not recommended to evaluate unbalanced MLGM. These findings are different from balanced MLGM study, where Hsu et al. (2019) showed that all χ^2^ test statistics, χ^2^_PS_B_, χ^2^_PS_W_, χ^2^_T_S_COV_, and χ^2^_T_S_MEAN_, can detect the misspecified balanced MLGM when NG is larger than 200 and GS is larger than 20. RMSEA_PS_B_ and RMSEA_PS_W_ are recommended to researchers to evaluate unbalanced MLGM with similar conditions used in the study. Based on our outcomes, RMSEA_T_S_COV_ is not recommended to evaluate unbalanced MLGM. Researchers are recommended to use RMSEA_T_S_MEAN_ when the sample size is large enough (NG > 50 and unbalanced GS > 5/15). Except TLI_T_S_COV_, all the other CFI-related fit indices and TLI-related fit indices are recommended to evaluate unbalanced MLGM. Researchers are recommended to use TLI_T_S_COV_ when the sample size is large enough (NG > 50 and unbalanced GS > 5/15). SRMR_B_, SRMRw, and SRMR_T_S_MEAN_ are recommended to evaluate unbalanced MLGM. The means of SRMR_T_S_COV_ had approached values did not approach the values indicative of good model fit (i.e., <0.08) under all simulation conditions.

Our results differed from previous level-specific and target-specific fit indices study conducted under balanced MLGM. Hsu et al. (2019) balanced MLGM study concluded that RMSEA_PS_B_, RMSEA_PS_W_, RMSEA_T_S_COV_, CFI_T_S_COV_, TLI_T_S_COV_, RMSEA_T_S_Mean_, and TLI_T_S_ Mean_ were significantly influenced by NG and should not be used. Hsu et al. (2019) also concluded that CFI_PS_B_, TLI_PS_B_, RMSEA_PS_W_, CFI_T_S_COV_, TLI_T_S_COV_, RMSEA_T_S_Mean_, CFI_T_S_Mean_, and TLI_T_S_ Mean_ were influenced by different GSs. A plausible explanation for the differences could be due to the unbalanced GS. Our study simulated three unbalanced GS, 5/15, 10/30, and 25/75. The balanced GS used in Hsu et al. (2019)’s design are: 5, 10, and 20. Hox and Maas (2001) investigated the performance of a MCFA (multilevel confirmatory factor model) on unbalanced data. The results indicated that unbalanced data had impact on the accuracy of model estimation of the between level model. As unbalanced MLGM have different between-level and within-level at each time point, the unbalanced data have an large influence on the model estimation of both levels.

Based on the results, we recommend researchers to collect at least 50 for NG, regardless of the GS and if the MLGM design is unbalanced or balanced. When the NG is small, the amount of the sampling errors presents with the between-level related specific model fit indices will increase due to that small samples might commit a Type II error for χ^2^_PS_B_. The large sampling error causes some between-level or target-specific fit indices for the between-covariance or between-mean structure to fail to identify a correctly specified between-level model. In this way, between-level related specific model fit indices, χ^2^_PS_B_, CFI_PS_B_, TLI_PS_B_, RMSEA_PS_B_, and SRMR_B_, require NG at least to be large enough (e.g., NG > 50) to be able to identify correctly specific MLGM with unbalanced design. However, if the NG cannot be at least 50 NG, RMSEA_PS_B_, SRMRB and SRMR_W_ are not recommended. Based on our results, χ^2^_PS_B_ and χ^2^_PS_W_ are not recommended with unbalanced MLGM designs when researchers have a NG smaller than 50. If researchers have moderate NG (around 100 cases), we recommend researchers to collect a GS larger than 5/15.

The combination of NG and unbalanced GS determine the total sample size in MLGMs and may also influence the performance level-specific and target-specific model fit indices. Pervious research illustrated that total sample size highly influenced the values of fit indices because of the issue of total sample size discrepancy, that is, the difference between a sample covariance matrix and the covariance matrix of the population (Marsh et al., 2014; Wu et al., 2009; Wu and West, 2010). When the total sample size is small, the discrepancy between the sample covariance matrix and the population covariance matrix will increase, and the discrepancy between a sample covariance matrix and covariance matrix reproduced by a correctly specified model will also increase. The large discrepancy will cause the fit indices to indicate poor fit for a correctly specified model. In contrast to the between-model evaluation, both NG and unbalanced GS jointly determine the sample size of the within-level model and influence the performance of within-level specific model fit indices. As for the effect of unbalanced GS, χ^2^_PS_W_ failed to identify correctly specified within-level model when the unbalanced GS was small (e.g., unbalanced GS < 10/30). When the unbalanced GS is small, the amount of the sampling errors calculated in within-level specific model fit indices increases. Based on the findings from our analysis and from previous research, we highly recommend applied researchers to collect average GS of 20 and consider NG when evaluating within-level related specific model fit indices, regardless of if the MLGM design is unbalanced or balanced.

### Recommended fit indices for each data type

**Table:** 

Fit indices		Sample size large enough	All condition for unbalanced MLGM	Not recommend for unbalanced MLGM
Level-specific fit indices	χ^2^_PS_B_	√ (NG > 100 and unbalanced GS > 10/30)		
	χ^2^_PS_W_	√ (NG > 100 and unbalanced GS > 10/30)		
	RMSEA_PS_B_		√	
	RMSEA_PS_W_		√	
	CFI_PS_B_		√	
	CFI_PS_W_		√	
	TLI_PS_B_		√	
	TLI_PS_W_		√	
	SRMR_B_		√	
	SRMR_W_		√	
Target-specific fit indices	χ^2^_T_S_COV_			√
	χ^2^_T_S_Mean_			√
	RMSEA_T_S_COV_			√
	RMSEA_T_S_Mean_	√ (NG > 50 and unbalanced GS > 5/15)		
	CFI_T_S_COV_		√	
	CFI_T_S_Mean_		√	
	TLI_T_S_COV_	√ (NG > 50 and unbalanced GS > 5/15)		
	TLI_T_S_ Mean_		√	
	SRMR_T_S_COV_			√
	SRMR_T_S_Mean_		√	

## Limitations and future research direction

In the study, we set the coefficient of variation constant throughout different group sizes, meaning that conclusions are limited to only one degree of imbalance. Future study need to vary the degree of imbalance to check the influence of imbalance on the evaluation of model fit indices. Misspecifications for the between and within models were not modeled. As there is not yet literature informing researchers about the performance of different level-specific and target-specific model fit indices in unbalanced MLGM, a correctly specified MLGM was simulated to fill this gap as a first step. As misspecifications in MLGM are possible, this aspect deserves systematic investigation in future simulation studies. By investigating different misspecifications, we can study the indices’ sensitivity, which measures the extent to which specific fit indices could reflect the discrepancy between correctly specified models and misspecified hypothesized models. We expected desirable fit indices to demonstrate reasonable sensitivity to minor misspecifications and to be able to detect moderate misspecifications at both levels. Besides, in our study, we considered a few numbers of design factors. Other design factors, such as a different number of time points and trajectories, can be manipulated in future studies.

## Data availability statement

The raw data supporting the conclusions of this article will be made available by the authors, without undue reservation.

## Author contributions

FP: Writing – original draft. QL: Writing – review & editing.
